# Supplementary material for: Structural effects of the highly protective V127 polymorphism on human prion protein
Source: Commun Biol. 2020 Jul 29;3:402. doi: 10.1038/s42003-020-01126-6 (PMC7391680; doi:10.1038/s42003-020-01126-6)
Supplement: Supplementary file 1 — Supplementary Information [file 42003_2020_1126_MOESM1_ESM.pdf]

Supplementary information

Structural effects of the highly protective V127 polymorphism on human prion protein

Hosszu *et al.*

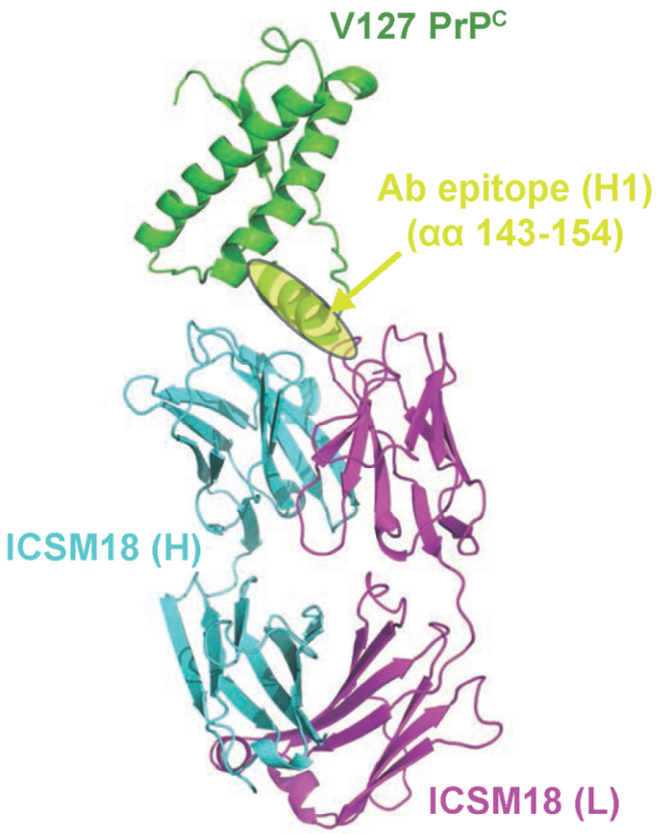

Supplementary Figure 1. The complex between recombinant V127/M129 PrP and ICSM-18 Fab as determined by X-ray crystallography

The secondary structure of the Fab-PrP complex is displayed in ribbon representation, with V127 PrP shown in green with the heavy and light chains of the Fab in cyan and magenta, respectively. The antibody epitope spans the whole of helix 1 (residues 143 – 154), and is remote from the site of the V127 and V129 polymorphisms, which are located near to, and within the PrP β-sheet. Co-crystallisation with the ICSM18 Fab fragment does not affect the PrP structures obtained[1,2].

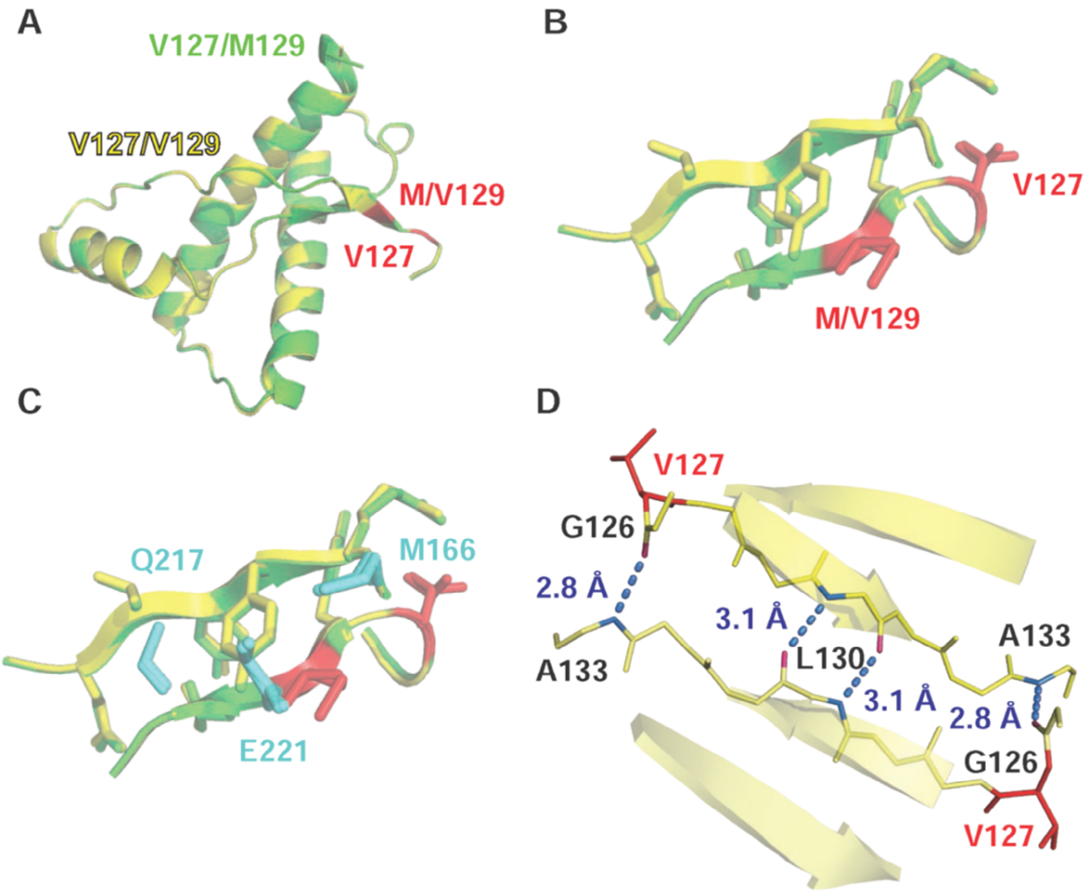

Supplementary Figure 2. Comparison of the secondary structure, side chain packing and intermolecular β-sheet contacts of V127 PrP with methionine and valine at residue 129

(A) *Superposition of secondary structure elements.* V127/M129 (pdb 6SV2) is shown in green and V127/V129 (pdb 6SUZ) in yellow. Residues 125–224 are shown, with residues 127 and 129 highlighted in red. The r.m.s. deviations for backbone heavy atoms within core structured regions (residues 128 – 131 (β-strand 1), 144 – 154 (α-helix 1), 160 – 164 (β-strand 2), 174 – 186 (α-helix 2), 202 – 220 (α-helix 3)) are less than 0.19 Å between the V129 variants.

(B) *Comparison of side chain packing in the PrP β-sheet, with the sidechains of residues 127 & 129 shown in stick representation.* The V129 sidechain is wedged in a hydrophobic pocket between Y163 and P165, with the M129 sidechain protruding from this to the protein surface. The bulkier M129 sidechain is accommodated with a small bulge in the strand (0.25 Å Cα displacement). The residue 129 polymorphism does not perturb the backbone or side-chain positions or hydrogen bonding of the β-sheet.

(C) *Amino acid side chain groups within 7 Å of the valine 129 sidechain in V127/V129 are shown in cyan stick representation.* Residues spatially close to residue 129 are also not perturbed by the polymorphism. The minor perturbations caused by the residue 129 methionine/valine polymorphism observed here are consistent with previous structural studies of the V129 polymorph[2,3].

(D) *Intermolecular β-sheet contacts in the V127/V129 PrP crystal.* The hydrogen bonds stabilising the V127/V129 PrP intermolecular β-sheet are shown as blue dotted lines between the amide and carbonyl groups of the denoted amino acids, with the corresponding distances in Å. The protective residue 127 polymorphism is shown in red. As with the V127/M129 PrP variant, an additional pair of hydrogen bonds (between G126 CO and A133 H^N) stabilises the intermolecular dimer interface, due to the change in backbone conformation caused by the incorporation of valine at residue 127.

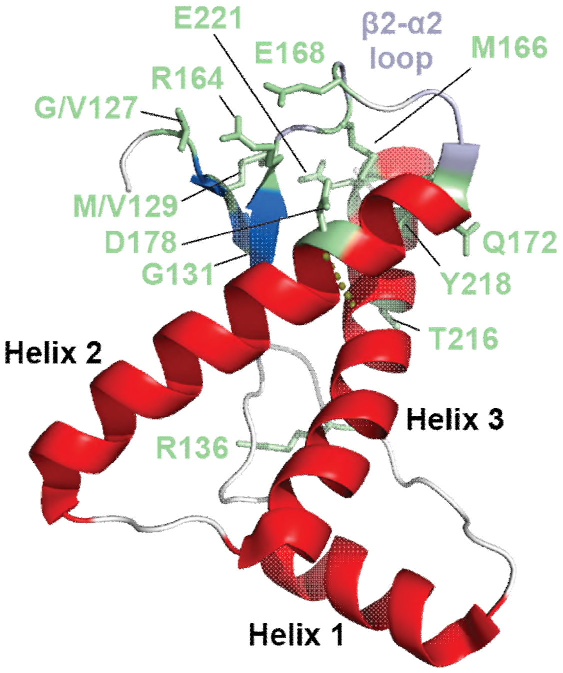

Supplementary Figure 3. PrP[C] secondary structure and residues which experience altered ms dynamics due to the V127polymorphism

Cartoon representation of the V127/M129 PrP crystal structure, with α-helices coloured red, β-strands blue, and the β2-α2 loop in light blue. The protective polymorphisms at residues 127 and 129, the disease-associated residue D178 and those residues which experience altered ms dynamics due to the V127 polymorphism are shown as stick representations (see Figure 4). The disulphide bond linking the side chains of cysteine 179 and 214 is shown as a yellow dashed line between the Cα positions of those residues.

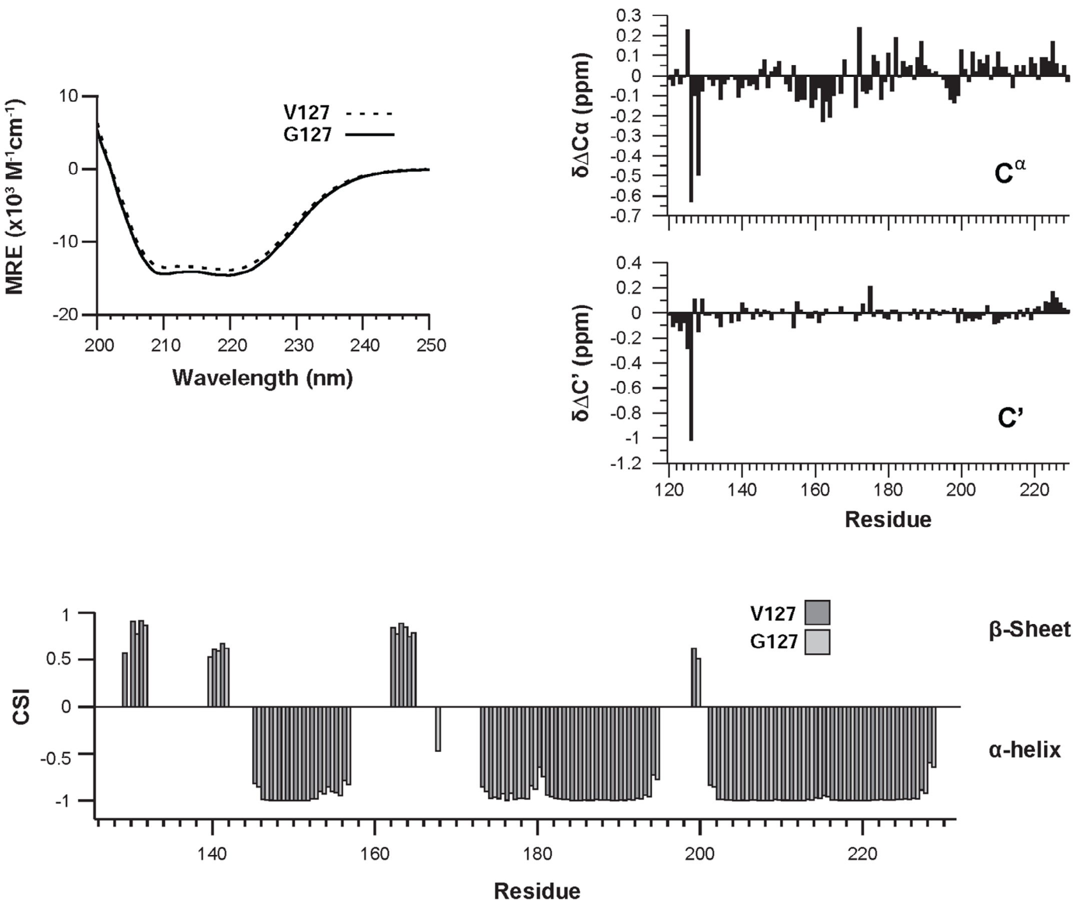

Supplementary Figure 4. V127/M129 crystal structure accurately reflects structure in solution

(A) *Far UV CD spectra of human wild-type G127/M129 PrP, and the protective V127/M129 PrP variant.* These display double minima at 208 & 222 nm, conventional indicators of α-helical structure. Spectra were compiled from an average of 50 scans. The Far-UV CD spectra for all PrP variants are very similar (see also Supplementary Figure 5), confirming the overall lack of structural perturbations associated with the V127 and V129 polymorphisms.

(B) *Chemical shift differences of Cα and C' nuclei between wild-type G127/M129 and variant V127/M129 PrP.* Cα and C' chemical shifts, which are particularly diagnostic of changes in backbone conformation[4,5], are very similar in both variants, and consistent with the relative lack of structural perturbation observed in the V127 PrP crystal. The largest changes immediately surround the site of the polymorphism, and are caused in part by the change in chemical composition, but significantly also reflect the considerable change in backbone conformation induced by the conformational restriction introduced by the valine substitution at residue 127 (see Figure 1(B)).

(C) *Chemical Shift Index (CSI) of human PrP (V127/M129 and G127/M129) calculated by TALOS*[5], showing regions predicted to be β-sheet (values above line) and α-helix (values below line). The solution structures of both PrP variants closely mirror their respective crystal structures, and support the notion that the crystal structures accurately reflect solution structure.

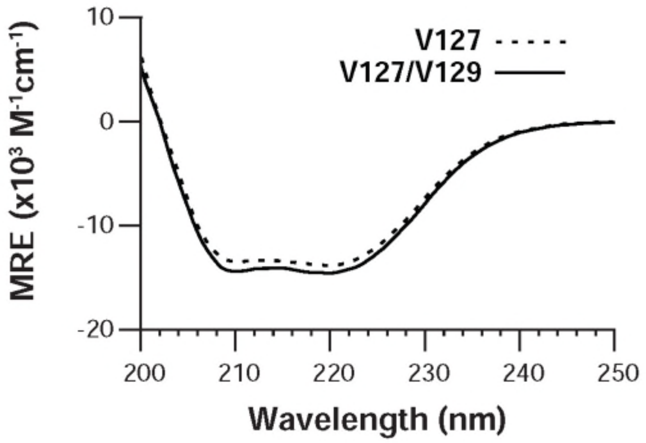

Supplementary Figure 5. Far UV CD spectra of human V127/M129 and V127/V129 PrP

These support the lack of structural perturbation observed in the PrP crystal structures caused by the protective valine polymorphs. CD spectra were compiled from an average of 50 scans.

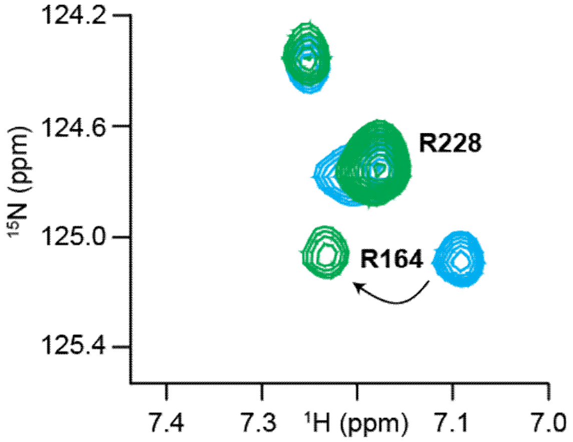

Supplementary Figure 6. Perturbation of the side-chain environment of arginine 164 (R164) induced by V127

Overlay of ^{1}H-^{15}N HSQC spectra of wild-type G127/M129 PrP (cyan) and V127/M129 PrP (green). The region of the spectra shown highlights resonances which arise from the Nε nuclei of arginine sidechain groups, and shows the distinct chemical shift perturbation of R164 Nε in V127/M129 PrP, confirming the alteration in its position observed in the V127 crystal structures.

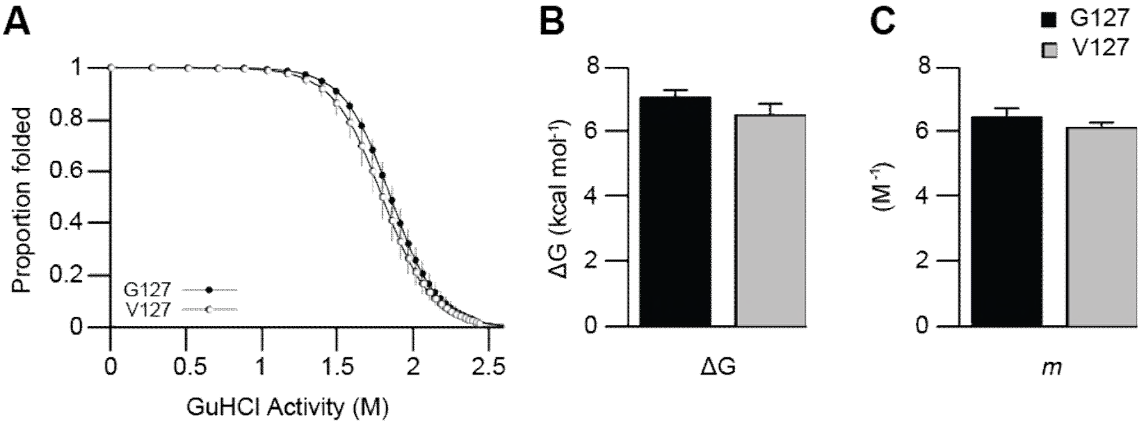

Supplementary Figure 7. V127 polymorphism does not significantly affect PrP stability

(A) *Equilibrium denaturation curves of wild-type G127/M129 and V127/M129 PrP.* Both proteins denature in a single co-operative transition, consistent with solely the folded and unfolded states populated. The fraction of the folded state molecules folded was determined by normalised amide CD absorption at 222 nm and plotted against denaturant activity (see Methods Section). Each measurement was repeated 3 times, with std. errors of each point displayed. The lines superimposed on the data are fits to a two-state model of folding (equation 1), as described in the Methods section.

(B) *The free energy change (ΔG) of the equilibrium unfolding transition* calculated using equation 1, which describes the two-state model of folding, are the same within error for G127/M129 and V127/M129 PrP (see Supplementary Table 2).

(C) *m values for G127/M129 and V127/M129 PrP, derived from the above equilibrium denaturation curves.* The *m* values, describe the sensitivity of the folded / unfolded state equilibrium to denaturant, and reflect the increase in solvent exposure of the hydrophobic core as the protein unfolds[6,7] (See Supplementary Table 2). These are also the same within error for both G127/M129 and V127/M129 PrP.

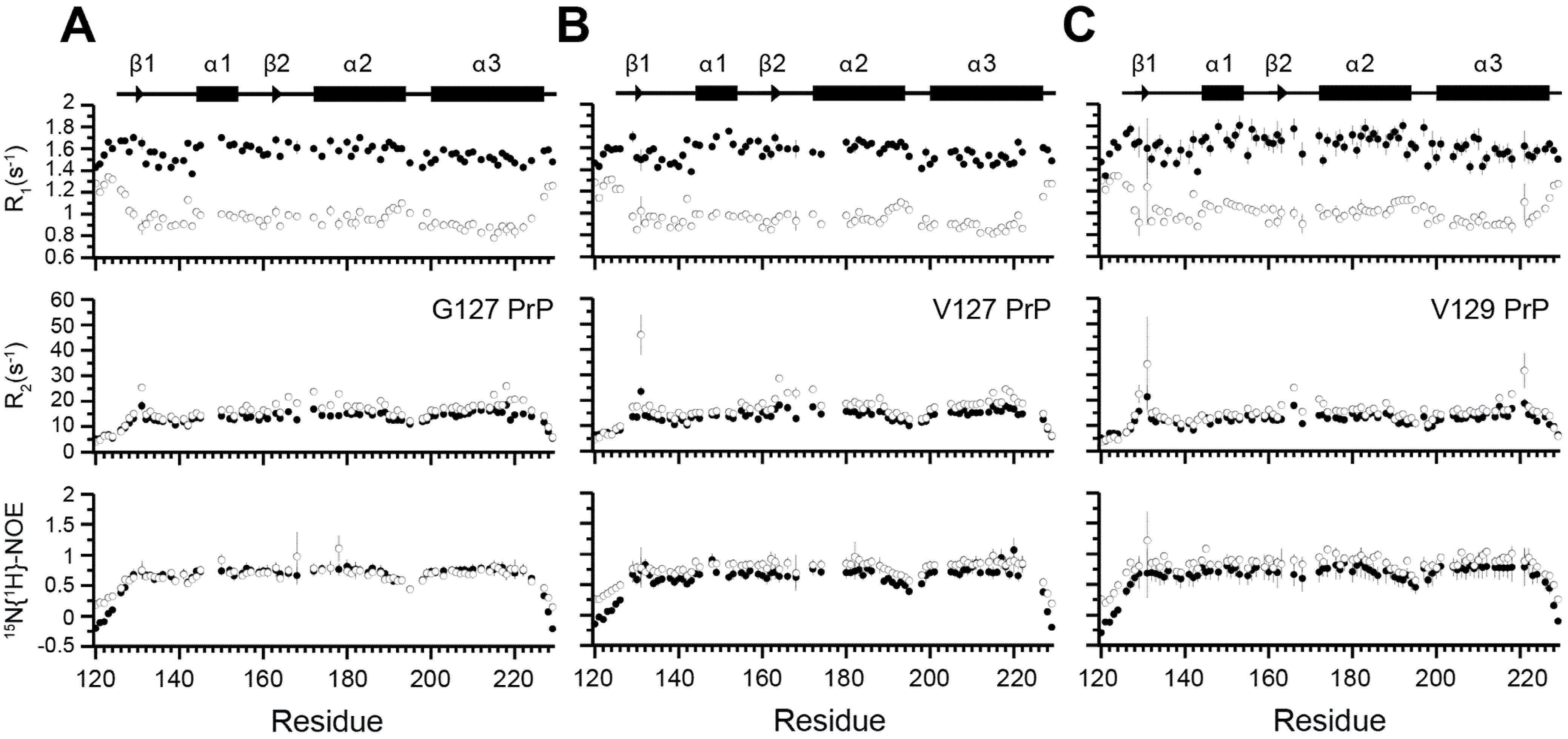

Supplementary Figure 8. ^{15}N spin relaxation data used to assess the dynamics of the PrP variants

Longitudinal ($R_1 = 1/T_1$) and transverse ($R_2 = 1/T_2$) ^{15}N relaxation rates, and $^{15}N\{^1H\}$-NOE enhancements of (A) G127 (G127/M129), (B) V127 (V127/M129) and (C) V129 (G127/V129) PrP at 500 MHz (filled circles) and 800 MHz (open circles). The R_2 rates for a number of residues in the structured region of PrP, for example G131, are considerably greater than the mean values for the rest of the structured domain. Residues with large R_2 rates are typically associated with slow conformational rearrangements in the millisecond to microsecond timescale. Regions of human PrP secondary structure are denoted as bars at the top of the figure[3,8].

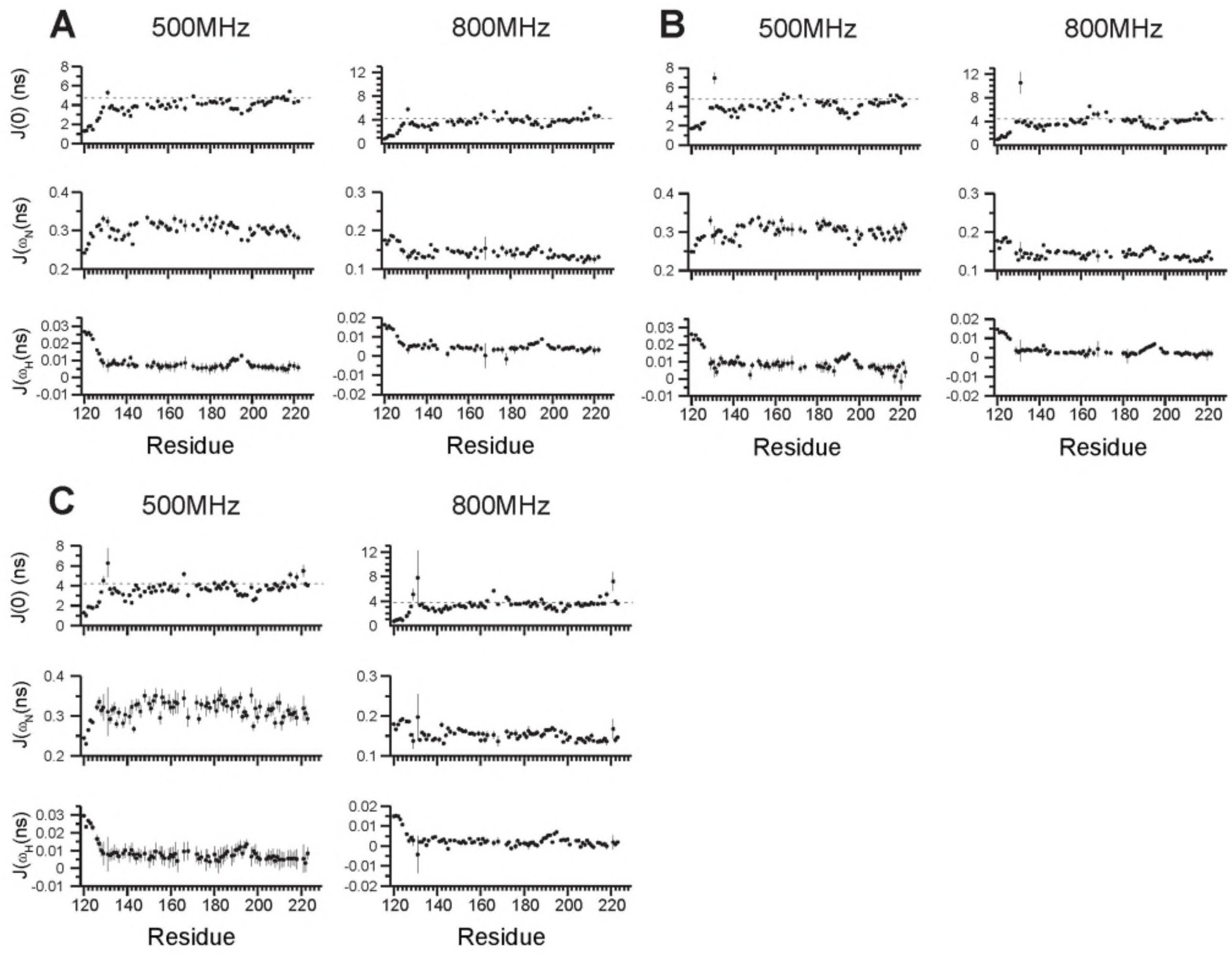

Supplementary Figure 9. Reduced spectral density functions describing the amplitude of different timescale motions in the various PrP variants

$J(0)$, $J(\omega_N)$ and $J(\omega_H)$ spectral densities for (A) G127/M129, (B) V127/M129 and (C) G127/V129 PrP at 500 MHz and 800 MHz. The spectral density function quantifies the amplitude of motion at a particular frequency (ω), where ω_N and ω_H are the ^{1}H and ^{15}N Larmor frequencies respectively. Unlike $J(\omega_H)$ and $J(\omega_N)$, $J(0)$, the spectral density at zero frequency, incorporates slow milli-second to microsecond timescale motions. $J(0)$ values significantly greater than the majority of the protein are strongly indicative of ms timescale conformational rearrangements, for example residue G131. The dotted lines in the $J(0)$ graphs are set at two standard deviations greater than the mean $J(0)$ spectral density for the N-terminus (residues 200-210) of helix 3 of the respective variants.

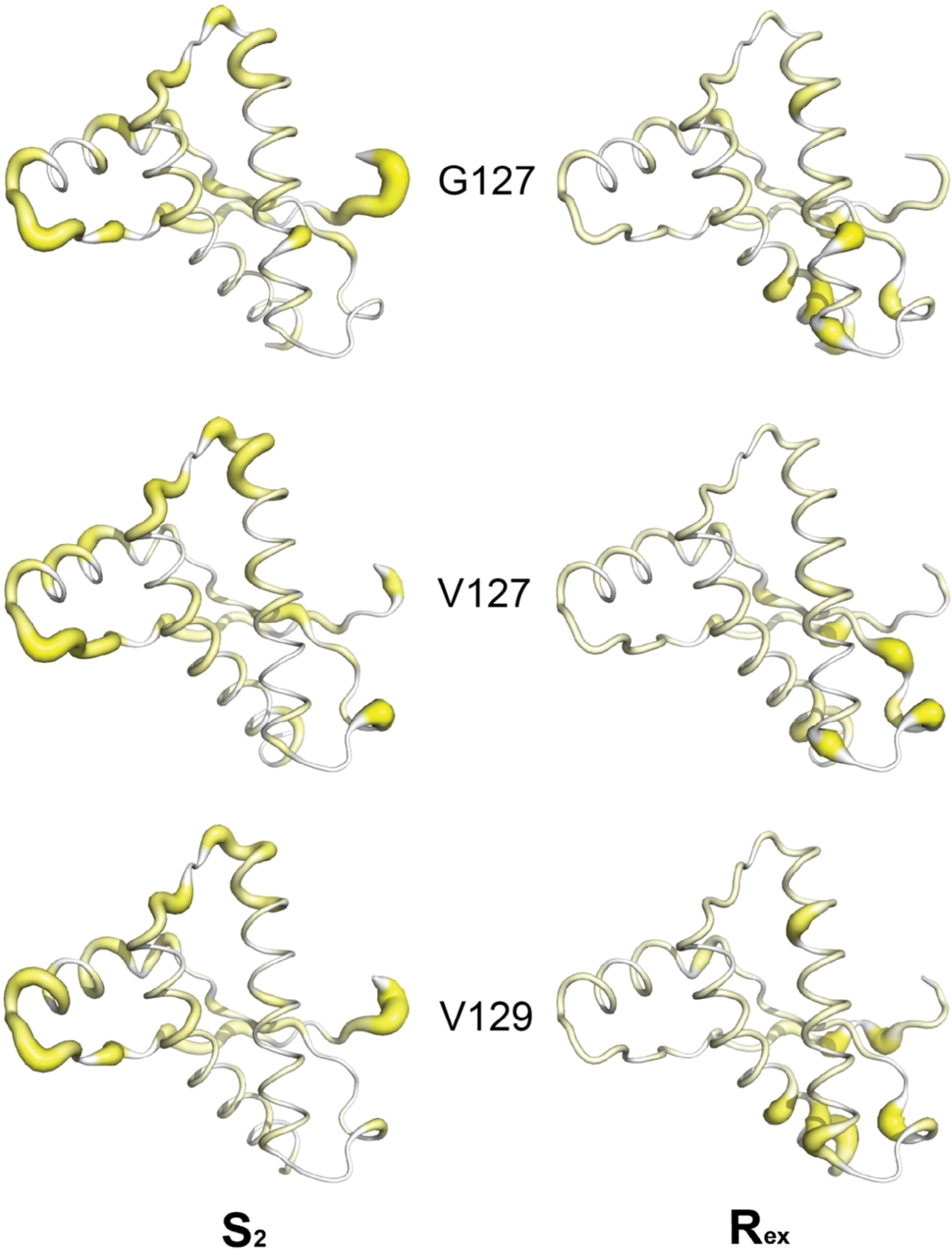

Supplementary Figure 10. Degree of order (S^2) and ms conformational dynamics (R_{ex}) in the PrP polymorphs

S^2 and R_{ex} values calculated by the *Relax* "d'Auvergne" *Modelfree* analysis[9], illustrated on the 3D structure of G127 (G127/M129), V127 (V127/M129) and V129 (G127/V129) PrP, by colour (white → yellow) and width of the protein backbone. Residues for which data was not available are coloured white. Large S^2 values indicate a greater degree of disorder and flexibility, for instance at the PrP N-terminus, and the loop linking helices 2 and 3. ms conformational dynamics are clustered in the β-sheet, the β2-α2 loop, and the C-terminus of helix 3.

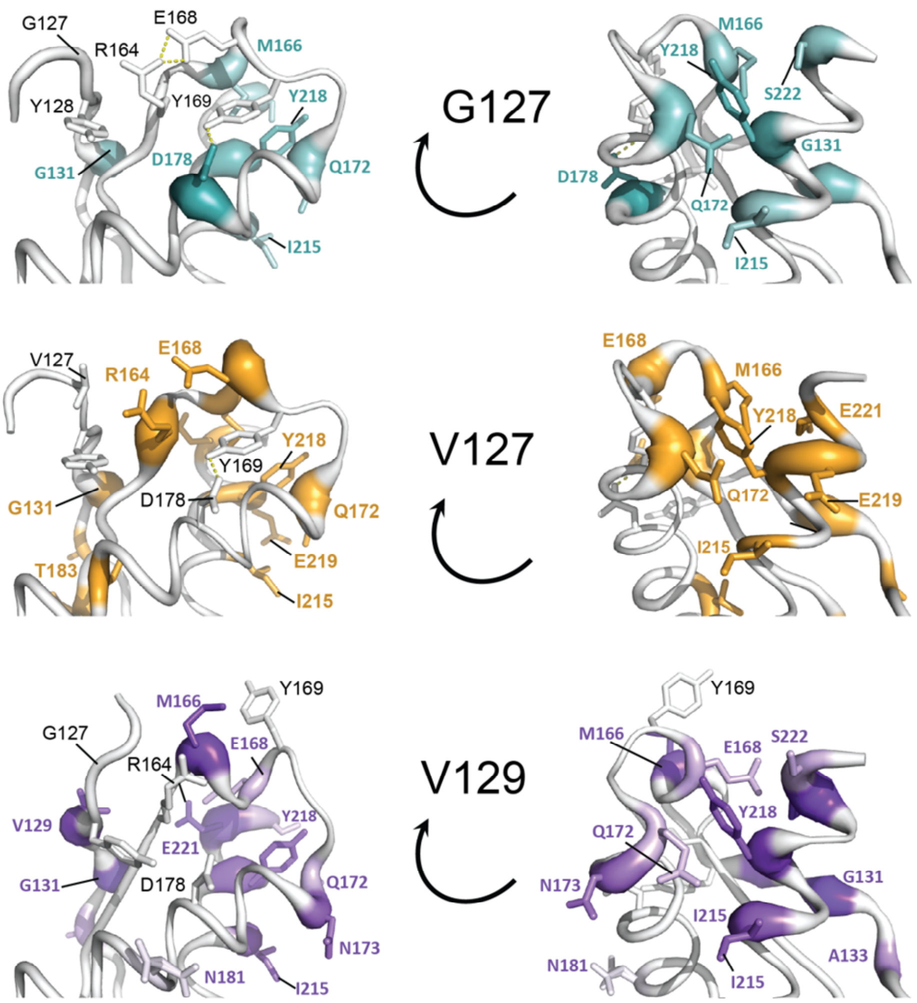

Supplementary Figure 11. Regions of PrP which experience slow conformational exchange (R_{ex}) motions in PrP^C, and hydrogen-bonding of Y169

The sidechains of residues which experience slow conformational exchange (R_{ex}) motions in the various PrP polymorphs, as calculated by the *Relax* “d’Auvergne” *Modelfree* analysis[10,11] are coloured, with varying width of backbone according to the degree of R_{ex} conformational exchange[2,8]. The structures of the PrP molecules are rotated in the right-hand column of molecules. The location of key sidechain groups and hydrogen bonding interactions are shown.

The positioning of the Y169 sidechain within the protein core is consistently maintained in a wide range of crystal structures, with the notable exception of G127/V129 human PrP^C (pdb **3HAK** - shown here[2]) and also the disease associated D178N form of human PrP^C (pdb **3HEQ**)[2]. In G127/V129 PrP^C, the aromatic side-chain of Y169 is solvent-exposed, with the side chain of D167 occupying its position and interacting with the side-chain of D178. In this structure, there is an extensive hydrogen-bonding network involving the side-chains of D178 – D167 – R164 and the mainchain amide of G126 which stabilises this loop conformation, in addition to the hydrogen-bonding within the loop itself[2]. The aromatic side chain of Y169 also interacts with the side chains of residues 166, 175, 218, 221, and 225, which also experience slow conformational exchange dynamics observed here. The residue 129 sidechain itself is predominantly solvent exposed, and substitution of the smaller valine sidechain does not cause any local conformational changes[2,3]. The residue 129 polymorphism thus also appears to exert some of its influence via the structure of the adjacent β2-α2 loop. The altered conformational dynamics of residues in the β2-α2 loop, and residue 178 support this idea, although the conformational dynamics of the β-sheet itself is altered by the valine 129 substitution itself, suggesting that the PrP β-sheet or residues in its immediate vicinity is also capable of adopting alternative low energy structures.

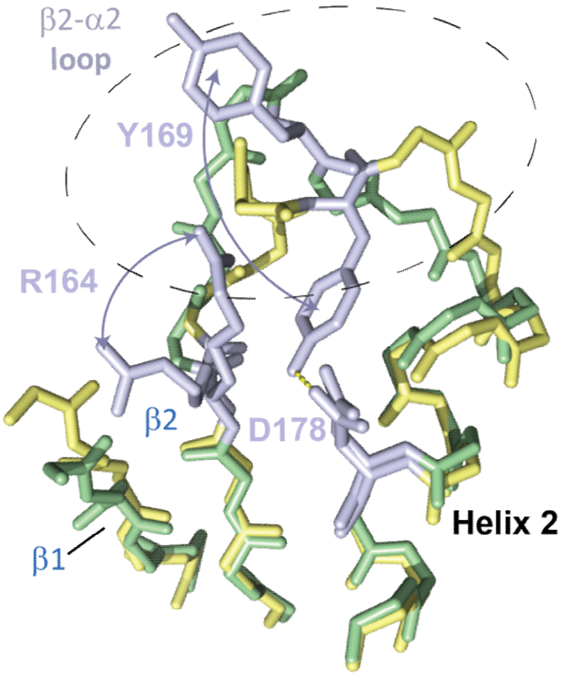

Supplementary Figure 12. 3_{10}-helix & type I β-turn β2-α2 loop conformations, and potential influence of the V127 polymorphism on their conformational distribution

Residue interactions in the 3_{10}-helical & type I β-turn conformations of the β2-α2 loop (R164 – Q172) seen in crystal structures of human PrP, (pdb **4N90** (1.5 Å resolution; yellow)[12] and pdb **3HES** (2.0 Å resolution; green)[2]). Mainchain atoms from residues in the vicinity of the first β-strand (G127 - G131), second β-strand (V161 - Y163) and parts of helix 2 (N173 – N181) are also shown, along with the sidechains of residues R164, Y169 and D178, which are in light blue. The hydrogen bonding between the sidechains of Y169 and D178 is removed when Y169 switches to its alternative solvent-exposed position.

The β2-α2 loop can adopt a 3_{10}-helical or β-turn conformation[13,14]. In the 3_{10}-helical conformation, the Y169 aromatic ring packs as part of the protein core, between helix 2 and the start of the β2-α2 loop, whereas in the β-turn conformation, it is translocated more than 13 Å (atom OH) and becomes fully solvent exposed. Increased solvent exposure of Y169 has been proposed to be associated with human prion disease mutations[15]. In the 3_{10}-helical conformation, the sidechain group of Y169 is in a position to directly influence the dynamics of residue 178 and packs closely against the amide of F175. This results in the NH group of F175 experiencing a strong ring current shift from the close "face-on" association with phenolic group of Y169[12]. Using *SHIFTX2*[16] and these pdb structures, the calculated difference in chemical shift for the NH group of F175 is the largest in the protein (Δδ H^N = 1.44 ppm) (See Supplementary Note 1). Hence, this resonance would be particularly susceptible to line-broadening from exchange between these conformers on a ms timescale. In the NMR spectra of all PrP^c variants studied here, the NH group of F175 is line-broadened beyond detection, whereas resonances from adjacent residues (Q172 – N174 and V176 onwards) are observable. Indeed, of the other four residues with resonances broadened beyond detection in HSQC spectra, three have calculated ^{1}H chemical shift differences greater than 0.8 ppm between structures 4N9O and 3HES (Δδ H^N Y169 = 0.88 ppm, Figure70 = 1.39 ppm, N171 = 1.16 ppm). The remaining NH resonance broadened beyond detection (D167), has a small, calculated chemical shift difference in H^N resonance between structures 4N9O and 3HES (Δδ H^N = 0.03 ppm), but the largest calculated chemical shift difference in ^{15}N resonance (Δδ N^H = 7.21 ppm) of the entire protein. Hence, the structures 4N9O and 3HES are good representatives of the conformations underlying this exchange process.

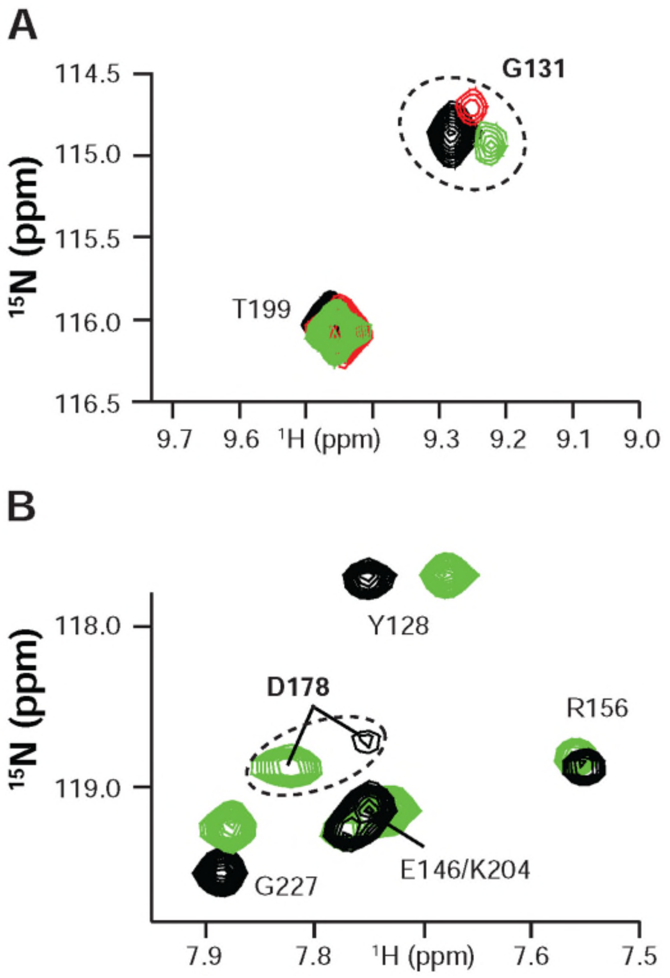

Supplementary Figure 13. Line-broadening of NMR signals in PrP polymorphs

Overlay of ^{1}H-^{15}N HSQC spectra of G127/M129 (black), V127/M129 (red), and G127/V129 (green) PrP.

(A) Reduced signal intensity of G131 in V127/M129 and G127/V129 HSQC spectra relative to G127/M129 PrP. The reduction is due to line-broadening of the NMR signal caused by ms conformational dynamics, as described in the text.

(B). Reduced signal intensity of D178 in G127/M129 PrP HSQC spectra relative to G127/V129 PrP. HSQC signals from residues V127 and D178 are heavily overlapped in V127/M129 PrP (red) HSQC spectra and are not overlaid in panel (B) for clarity.

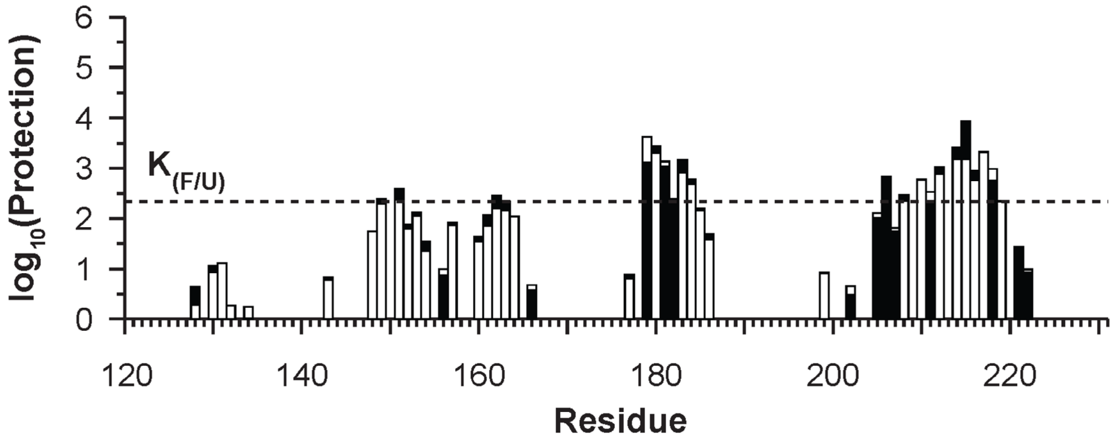

Supplementary Figure 14. Hydrogen exchange protection; effect of the V127 polymorphic mutation on the stability of PrP secondary structure elements

Amide protection factors (k_{ex}/k_{int}) in G127/M129PrP (White filled bars) and V127/M129 PrP (Black filled bars). Residues with measurable protection are shown, the remainder undetermined due to either peak overlap or lack of measurable protection. The protection factors for the core region of PrP^C are equal to the equilibrium constant between the folded (F) and unfolded (U) states of the protein ($K_{(F/U)}$)[17], indicating that PrP must fully unfold for its core residues to exchange with the solvent. Regions of highest protection surround the disulphide bond that links helices 2 and 3 at residues 179 and 214, and which forms residual structure in the unfolded state of the protein. The stability of the PrP core and secondary structure elements of both polymorphs are very similar.

	FAB18 – V127/M129 PrP (6SV2.pdb)	FAB18 – V127/V129 PrP (6SUZ.pdb)
**Data collection**		
Space group	$P6_322$	$P6_322$
Cell dimensions		
a, *b*, *c* (Å)	128.08, 128.08, 135.64	127.95, 127.95, 135.80
α, β, γ (°)	90, 90, 120	90, 90, 120
Resolution (Å)	58-2.3 (2.38-2.30) *	110.8-2.5(2.6-2.5)
Rpim	0.099 (1.224)	0.063 (0.464)
$I / \sigma I$	28.6 (2.9)	10.9 (2.6)
Completeness (%)	91.6 (92.5)	99.4 (99.6)
Redundancy	4.2 (4.1)	5.9 (5.9)
**Refinement**		
Resolution (Å)	57.9-2.3	111-2.5
No. reflections	24982	21923
R_{work} / R_{free}	0.20 / 0.25	0.16 / 0.21
No. atoms		
Protein	4037	4078
Ligand/ion	5	5
Water	63	66
B-factors		
Protein	47.6	57.3
Ligand/ion	24.6	36.5
Water	33.4	39.3
R.m.s. deviations		
Bond lengths (Å)	0.015	0.018
Bond angles (°)	1.735	1.882

*Values in parentheses are for highest-resolution shell.

Supplementary Table 1. Data collection and refinement statistics (molecular replacement)

Polymorphism	ΔG (kcal mol^{-1})	ΔΔG (kcal mol^{-1})	*m* (M^{-1})	Mid-point (D_m) (molar activity)
G127/M129	-7.06 ± 0.22	-	-6.46 ± 0.30	1.86 ± 0.01
V127/M129	-6.51 ± 0.34	0.55 ± 0.40	-6.13 ± 0.14	1.82 ± 0.02
V127/V129	-6.40 ± 0.06	0.66 ± 0.23	-5.85 ± 0.05	1.85 ± 0.01

Supplementary Table 2. Thermodynamic parameters of PrP β-sheet polymorphisms

The free-energy for folding (ΔG), degree of destabilisation (ΔΔG), and the midpoint of the equilibrium unfolding transition (D_m) for wild-type G127 PrP and variants V127 and V127/V129 were calculated by using a 2-state model from GuHCl-induced equilibrium unfolding transitions (See Supplementary Figure 7), monitored using the CD signal at 222 nm. The folding midpoint (D_m) is expressed as molar denaturant activity, derived from GuHCl concentration as described in the Methods.

Supplementary Note 1: ShiftX2 Analysis

Backbone and side chain ^{1}H, ^{13}C and ^{15}N chemical shifts were predicted using *SHIFTX2*[16] using 4N90 and 3HES pdb entries and the default input options (pH 5/298 K).

Supplementary References

1 Haire, L. F. et al. The crystal structure of the globular domain of sheep prion protein. *J Mol. Biol.* **336**, 1175-1183 (2004).

2 Lee, S. et al. Conformational diversity in prion protein variants influences intermolecular beta-sheet formation. *EMBO J.* **29**, 251-262 (2010).

3 Hosszu, L. L. P. et al. The residue 129 polymorphism in human prion protein does not confer susceptibility to CJD by altering the structure or global stability of PrP^C. *J. Biol. Chem.* **279**, 28515-28521 (2004).

4 Wishart, D. S., Sykes, B. D., & Richards, F. M. Relationship between nuclear magnetic resonance chemical shift and protein secondary structure. *J Mol. Biol.* **222**, 311-333 (1991).

5 Cornilescu, G., Delaglio, F., & Bax, A. Protein backbone angle restraints from searching a database for chemical shift and sequence homology. *J. Biomol. NMR* **13**, 289-302 (1999).

6 Pace, C. N. The stability of globular proteins. *CRC Crit Rev Biochem* **3**, 1-43 (1975).

7 Parker, M. J., Spencer, J., & Clarke, A. R. An integrated kinetic analysis of intermediates and transition states in protein folding reactions. *J Mol Biol* **253**, 771-786 (1995).

8 Antonyuk, S. V. et al. Crystal structure of human prion protein bound to a therapeutic antibody. *Proc. Natl. Acad. Sci. USA* **106**, 2554-2558 (2009).

9 Lipari, G. & Szabo, A. Model-Free Approach to the Interpretation of Nuclear Magnetic-Resonance Relaxation in Macromolecules .1. Theory and Range of Validity. *J. Am. Chem. Soc.* **104**, 4546-4559 (1982).

10 d'Auvergne, E. J. & Gooley, P. R. Optimisation of NMR dynamic models I. Minimisation algorithms and their performance within the model-free and Brownian rotational diffusion spaces. *J Biomol NMR* **40**, 107-119 (2008).

11 d'Auvergne, E. J. & Gooley, P. R. Optimisation of NMR dynamic models II. A new methodology for the dual optimisation of the model-free parameters and the Brownian rotational diffusion tensor. *J Biomol NMR* **40**, 121-133 (2008).

12 Abskharon, R. N. et al. Probing the N-Terminal beta-Sheet Conversion in the Crystal Structure of the Human Prion Protein Bound to a Nanobody. *J Am Chem Soc* **136**, 937-944 (2014).

13 Damberger, F. F., Christen, B., Perez, D. R., Hornemann, S., & Wuthrich, K. Cellular prion protein conformation and function. *Proc Natl Acad Sci U S A* **108**, 17308-17313 (2011).

14 Christen, B., Damberger, F. F., Perez, D. R., Hornemann, S., & Wuthrich, K. Structural plasticity of the cellular prion protein and implications in health and disease. *Proc Natl Acad Sci U S A* **110**, 8549-8554 (2013).

15 Rossetti, G., Cong, X., Caliandro, R., Legname, G., & Carloni, P. Common Structural Traits across Pathogenic Mutants of the Human Prion Protein and Their Implications for Familial Prion Diseases. *J Mol Biol* **411**, 700-712 (2011).

16 Han, B., Liu, Y., Ginzinger, S. W., & Wishart, D. S. SHIFTX2: significantly improved protein chemical shift prediction. *J Biomol NMR* **50**, 43-57 (2011).

17 Hosszu, L. L. P. et al. Structural mobility of the human prion protein probed by backbone hydrogen exchange. *Nature Struct. Biol.* **6**, 740-743 (1999).
