# Supplementary material for: Structural effects of the highly protective V127 polymorphism on human prion protein
Source: Commun Biol. 2020 Jul 29;3:402. doi: 10.1038/s42003-020-01126-6 (PMC7391680; doi:10.1038/s42003-020-01126-6)
Supplement: Supplementary file 6 — Description of Additional Supplementary Files [file 42003_2020_1126_MOESM6_ESM.pdf]

Description of Additional Supplementary Files

File Name: **Supplementary Data 1**
Description: Source data underlying Figure 5

File Name: **Supplementary Data 2**
Description: Source data underlying Figure 6

File Name: **Supplementary Data 3**
Description: Source data underlying Figure 9
