# Supplementary material for: Structural effects of the highly protective V127 polymorphism on human prion protein
Source: Commun Biol. 2020 Jul 29;3:402. doi: 10.1038/s42003-020-01126-6 (PMC7391680; doi:10.1038/s42003-020-01126-6)
Supplement: Supplementary file 7 — Reporting Summary [file 42003_2020_1126_MOESM7_ESM.pdf]

nature research

Corresponding author(s): J.Collinge

Last updated by author(s): May 30, 2020

Reporting Summary

Nature Research wishes to improve the reproducibility of the work that we publish. This form provides structure for consistency and transparency in reporting. For further information on Nature Research policies, see Authors & Referees and the Editorial Policy Checklist.

Statistics

For all statistical analyses, confirm that the following items are present in the figure legend, table legend, main text, or Methods section.

n/a	Confirmed	
☐	☒	The exact sample size (n) for each experimental group/condition, given as a discrete number and unit of measurement
☐	☒	A statement on whether measurements were taken from distinct samples or whether the same sample was measured repeatedly
☐	☒	The statistical test(s) used AND whether they are one- or two-sided<br>*Only common tests should be described solely by name; describe more complex techniques in the Methods section.*
☒	☐	A description of all covariates tested
☒	☐	A description of any assumptions or corrections, such as tests of normality and adjustment for multiple comparisons
☐	☒	A full description of the statistical parameters including central tendency (e.g. means) or other basic estimates (e.g. regression coefficient) AND variation (e.g. standard deviation) or associated estimates of uncertainty (e.g. confidence intervals)
☐	☒	For null hypothesis testing, the test statistic (e.g. F, t, r) with confidence intervals, effect sizes, degrees of freedom and P value noted<br>*Give P values as exact values whenever suitable.*
☒	☐	For Bayesian analysis, information on the choice of priors and Markov chain Monte Carlo settings
☒	☐	For hierarchical and complex designs, identification of the appropriate level for tests and full reporting of outcomes
☒	☐	Estimates of effect sizes (e.g. Cohen's d, Pearson's r), indicating how they were calculated

Our web collection on statistics for biologists contains articles on many of the points above.

Software and code

Policy information about availability of computer code

Data collection	Topspin (v 3.2) (Bruker)
Data analysis	XDS (http://xds.mpimf-heidelberg.mpg.de/)<br>BLEND (http://www.ccp4.ac.uk/)<br>AIMLESS (http://www.ccp4.ac.uk/)<br>PHASER<br>COOT (v 0.8.2) (https://www2.mrc-lmb.cam.ac.uk/personal/pemsley/coot/)<br>REFMAC5 (http://www.ccp4.ac.uk/)<br>FELIX 2007 (Accelrys, San Diego)<br>CCPN Analysis (v. 2.3.1) (https://www.ccpn.ac.uk/)<br>RELAX (v 3.3.1) (https://www.nmr-relax.com/)<br>SHIFTX2 (http://www.shiftx2.ca/)<br>GRAFIT (v 5) (Erithacus software)<br>GNUplot (v5.2) (http://www.gnuplot.info/)

For manuscripts utilizing custom algorithms or software that are central to the research but not yet described in published literature, software must be made available to editors/reviewers. We strongly encourage code deposition in a community repository (e.g. GitHub). See the Nature Research guidelines for submitting code & software for further information.

Data

Policy information about availability of data

All manuscripts must include a data availability statement. This statement should provide the following information, where applicable:

- Accession codes, unique identifiers, or web links for publicly available datasets
- A list of figures that have associated raw data
- A description of any restrictions on data availability

The atomic coordinates for the crystal structures described in this paper have been deposited in the Brookhaven Protein Data Bank (https://www.rcsb.org/) (accession nos. 6SV2 & 6SUZ). The data that support the findings of this study are available from the corresponding author upon reasonable request.

Field-specific reporting

Please select the one below that is the best fit for your research. If you are not sure, read the appropriate sections before making your selection.

☒ Life sciences ☐ Behavioural & social sciences ☐ Ecological, evolutionary & environmental sciences

For a reference copy of the document with all sections, see nature.com/documents/nr-reporting-summary-flat.pdf

Life sciences study design

All studies must disclose on these points even when the disclosure is negative.

Sample size	No sample size calculations were performed. The sample size (n) of each experiment is provided in the corresponding figure captions in the main manuscript and supplementary information files. Sample sizes were chosen to support meaningful conclusions.
Data exclusions	No data were excluded from data analyses.
Replication	All in vitro folding experiments were replicated at least 3 times. In vitro fibrillisation assays were replicated 5 times. T1 & T2 NMR data each included one duplicate dataset. Replicate experiments were successful.
Randomization	In the reported experiments, each protein sample was identically-engineered. The work does not involve participant groups. T1 & T2 NMR data were recorded with randomised order of time increments.
Blinding	Investigators were not blinded during experimental measurements or data analysis.

Reporting for specific materials, systems and methods

We require information from authors about some types of materials, experimental systems and methods used in many studies. Here, indicate whether each material, system or method listed is relevant to your study. If you are not sure if a list item applies to your research, read the appropriate section before selecting a response.

Materials & experimental systems

n/a	Involved in the study	
☐	☒	Antibodies
☒	☐	Eukaryotic cell lines
☒	☐	Palaeontology
☒	☐	Animals and other organisms
☒	☐	Human research participants
☒	☐	Clinical data

Methods

n/a	Involved in the study	
☒	☐	ChIP-seq
☒	☐	Flow cytometry
☒	☐	MRI-based neuroimaging

Antibodies

Antibodies used	ICSM18-Fab (D-Gen Ltd., London, UK)
Validation	Antonyuk, S. V. et al. Crystal structure of human prion protein bound to a therapeutic antibody. Proc. Natl. Acad. Sci. USA 106, 2554-2558 (2009).
