# Supplementary material for: Structural effects of the highly protective V127 polymorphism on human prion protein
Source: Commun Biol. 2020 Jul 29;3:402. doi: 10.1038/s42003-020-01126-6 (PMC7391680; doi:10.1038/s42003-020-01126-6)
Supplement: Supplementary file 9 — PDB validation report 2 [file 42003_2020_1126_MOESM9_ESM.pdf]

Full wwPDB X-ray Structure Validation Report (i)

Sep 17, 2019 – 05:04 pm BST

PDB ID	:	6SV2
Title	:	Human prion protein (PrP) fragment 119-231 (G127V M129 variant) complexed to ICSM 18 (anti-Prp therapeutic antibody) Fab fragment
Deposited on	:	2019-09-17
Resolution	:	2.30 Å(reported)

This is a Full wwPDB X-ray Structure Validation Report.

This report is produced by the wwPDB biocuration pipeline after annotation of the structure.

We welcome your comments at *validation@mail.wwpdb.org*
A user guide is available at
https://www.wwpdb.org/validation/2017/XrayValidationReportHelp
with specific help available everywhere you see the (i) symbol.

CONFIDENTIAL VALIDATION REPORT

The following versions of software and data (see references (i)) were used in the production of this report:

MolProbity	:	4.02b-467
Mogul	:	1.8.0 (224370), CSD as540be (2019)
Xtriage (Phenix)	:	1.13
EDS	:	2.4
Percentile statistics	:	20171227.v01 (using entries in the PDB archive December 27th 2017)
Refmac	:	5.8.0158
CCP4	:	7.0 (Gargrove)
Ideal geometry (proteins)	:	Engh & Huber (2001)
Ideal geometry (DNA, RNA)	:	Parkinson et al. (1996)
Validation Pipeline (wwPDB-VP)	:	2.4

1 Overall quality at a glance (i)

The following experimental techniques were used to determine the structure:
X-RAY DIFFRACTION

The reported resolution of this entry is 2.30 Å.

Percentile scores (ranging between 0-100) for global validation metrics of the entry are shown in the following graphic. The table shows the number of entries on which the scores are based.

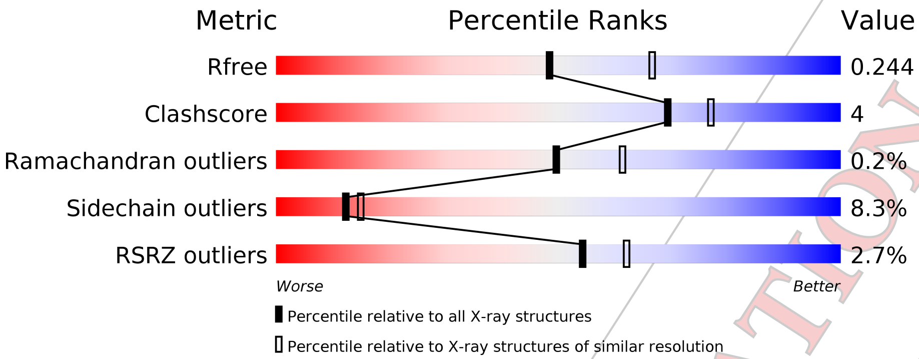

Metric	Whole archive (#Entries)	Similar resolution (#Entries, resolution range(Å))
R_{free}	111664	4477 (2.30-2.30)
Clashscore	122126	5072 (2.30-2.30)
Ramachandran outliers	120053	5022 (2.30-2.30)
Sidechain outliers	120020	5021 (2.30-2.30)
RSRZ outliers	108989	4374 (2.30-2.30)

The table below summarises the geometric issues observed across the polymeric chains and their fit to the electron density. The red, orange, yellow and green segments on the lower bar indicate the fraction of residues that contain outliers for >=3, 2, 1 and 0 types of geometric quality criteria. A grey segment represents the fraction of residues that are not modelled. The numeric value for each fraction is indicated below the corresponding segment, with a dot representing fractions <=5% The upper red bar (where present) indicates the fraction of residues that have poor fit to the electron density. The numeric value is given above the bar.

Mol	Chain	Length	Quality of chain
1	L	212	4% / 85% 13% ••
2	H	215	2% / 80% 13% • •
3	A	113	% / 73% 15% • 11%

CONFIDENTIAL VALIDATION REPORT

2 Entry composition (i)

There are 5 unique types of molecules in this entry. The entry contains 4127 atoms, of which 0 are hydrogens and 0 are deuteriums.

In the tables below, the ZeroOcc column contains the number of atoms modelled with zero occupancy, the AltConf column contains the number of residues with at least one atom in alternate conformation and the Trace column contains the number of residues modelled with at most 2 atoms.

- Molecule 1 is a protein called ICSM 18-ANTI-PRP THERAPEUTIC FAB LIGHT CHAIN.

Mol	Chain	Residues	Atoms					ZeroOcc	AltConf	Trace
			Total	C	N	O	S			
1	L	210	1626	1013	275	329	9	64	2	0

- Molecule 2 is a protein called ICSM 18-ANTI-PRP THERAPEUTIC FAB HEAVY CHAIN.

Mol	Chain	Residues	Atoms					ZeroOcc	AltConf	Trace
			Total	C	N	O	S			
2	H	208	1585	1007	256	317	5	50	3	0

- Molecule 3 is a protein called Major prion protein.

Mol	Chain	Residues	Atoms					ZeroOcc	AltConf	Trace
			Total	C	N	O	S			
3	A	101	848	527	146	166	9	21	0	0

There is a discrepancy between the modelled and reference sequences:

Chain	Residue	Modelled	Actual	Comment	Reference
A	127	VAL	GLY	conflict	UNP P04156

- Molecule 4 is SULFATE ION (three-letter code: SO4) (formula: O_4S).

CONFIDENTIAL VALIDATION REPORT

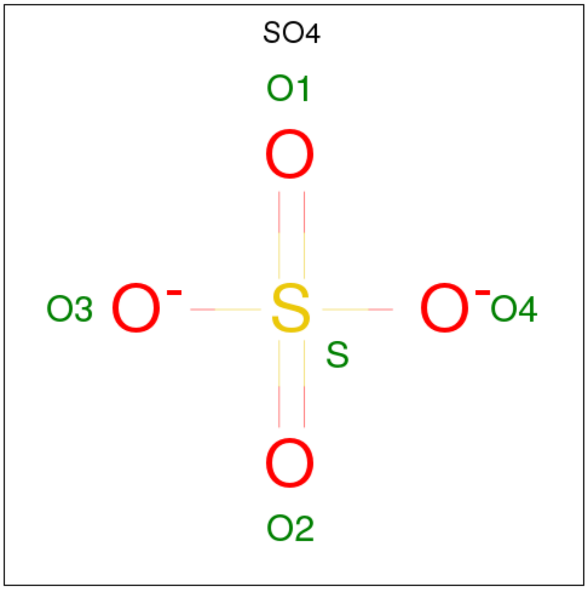

Mol	Chain	Residues	Atoms	ZeroOcc	AltConf
4	H	1	Total O S<br>5 4 1	0	0

- Molecule 5 is water.

Mol	Chain	Residues	Atoms	ZeroOcc	AltConf
5	L	25	Total O<br>25 25	0	0
5	H	20	Total O<br>20 20	0	0
5	A	18	Total O<br>18 18	0	0

CONFIDENTIAL VALIDATION REPORT

3 Residue-property plots ⓘ

These plots are drawn for all protein, RNA and DNA chains in the entry. The first graphic for a chain summarises the proportions of the various outlier classes displayed in the second graphic. The second graphic shows the sequence view annotated by issues in geometryand electron density. Residues are color-coded according to the number of geometric quality criteria for which they contain at least one outlier: green = 0, yellow = 1, orange = 2 and red = 3 or more. A red dot above a residue indicates a poor fit to the electron density (RSRZ > 2). Stretches of 2 or more consecutive residues without any outlier are shown as a green connector. Residues present in the sample, but not in the model, are shown in grey.

• Molecule 1: ICSM 18-ANTI-PRP THERAPEUTIC FAB LIGHT CHAIN

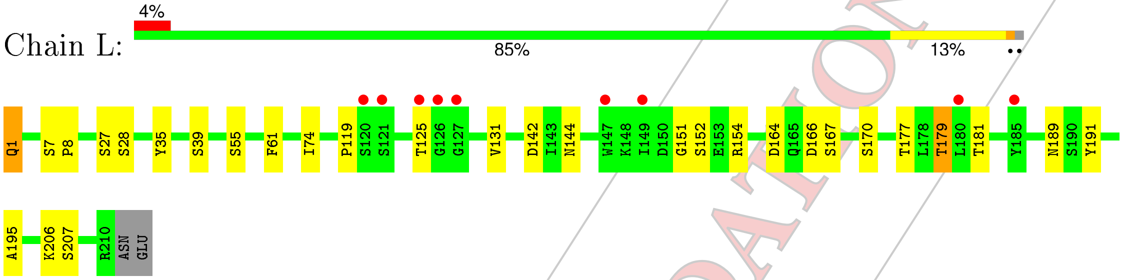

• Molecule 2: ICSM 18-ANTI-PRP THERAPEUTIC FAB HEAVY CHAIN

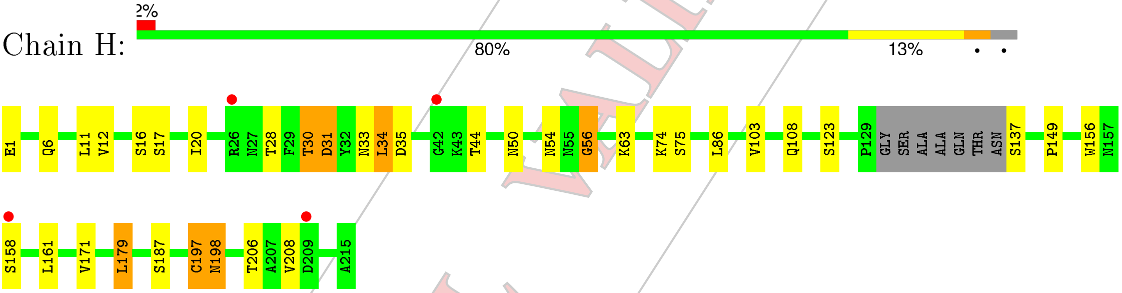

• Molecule 3: Major prion protein

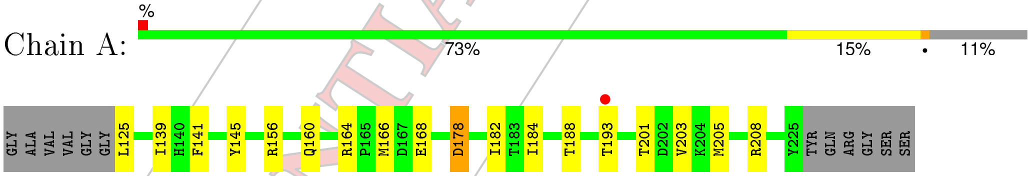

CONFIDENTIAL VALIDATION REPORT

4 Data and refinement statistics (i)

Property	Value	Source
Space group	P 63 2 2	Depositor
Cell constants<br>a, b, c, α, β, γ	128.08Å 128.08Å 135.64Å<br>90.00° 90.00° 120.00°	Depositor
Resolution (Å)	57.93 – 2.30<br>57.86 – 2.30	Depositor<br>EDS
% Data completeness<br>(in resolution range)	88.5 (57.93-2.30)<br>88.5 (57.86-2.30)	Depositor<br>EDS
R_{merge}	(Not available)	Depositor
R_{sym}	(Not available)	Depositor
$< I/\sigma(I) >$ [1]	2.40 (at 2.29Å)	Xtriage
Refinement program	REFMAC 5.7.0032	Depositor
R, R_{free}	0.200 , 0.251<br>0.196 , 0.244	Depositor<br>DCC
R_{free} test set	1362 reflections (5.17%)	wwPDB-VP
Wilson B-factor (Å^2)	37.9	Xtriage
Anisotropy	0.100	Xtriage
Bulk solvent k_{sol}(e/Å^3), B_{sol}(Å^2)	0.32 , 47.3	EDS
L-test for twinning[2]	$< \|L\| > = 0.49$, $< L^2 > = 0.32$	Xtriage
Estimated twinning fraction	No twinning to report.	Xtriage
F_o,F_c correlation	0.95	EDS
Total number of atoms	4127	wwPDB-VP
Average B, all atoms (Å^2)	47.0	wwPDB-VP

Xtriage's analysis on translational NCS is as follows: *The largest off-origin peak in the Patterson function is 2.82% of the height of the origin peak. No significant pseudotranslation is detected.*

[1] Intensities estimated from amplitudes.

[2] Theoretical values of $< |L| >$, $< L^2 >$ for acentric reflections are 0.5, 0.333 respectively for untwinned datasets, and 0.375, 0.2 for perfectly twinned datasets.

CONFIDENTIAL VALIDATION REPORT

5 Model quality (i)

5.1 Standard geometry (i)

Bond lengths and bond angles in the following residue types are not validated in this section: SO4

The Z score for a bond length (or angle) is the number of standard deviations the observed value is removed from the expected value. A bond length (or angle) with $|Z| > 5$ is considered an outlier worth inspection. RMSZ is the root-mean-square of all Z scores of the bond lengths (or angles).

Mol	Chain	Bond lengths		Bond angles	
		RMSZ	#$|Z|$ >5	RMSZ	#$|Z|$ >5
1	L	0.77	1/1673 (0.1%)	0.79	0/2270
2	H	0.76	0/1639	0.84	3/2246 (0.1%)
3	A	0.93	1/867 (0.1%)	0.96	4/1171 (0.3%)
All	All	0.80	2/4179 (0.0%)	0.85	7/5687 (0.1%)

Chiral center outliers are detected by calculating the chiral volume of a chiral center and verifying if the center is modelled as a planar moiety or with the opposite hand.A planarity outlier is detected by checking planarity of atoms in a peptide group, atoms in a mainchain group or atoms of a sidechain that are expected to be planar.

Mol	Chain	#Chirality outliers	#Planarity outliers
2	H	0	1

All (2) bond length outliers are listed below:

Mol	Chain	Res	Type	Atoms	Z	Observed(Å)	Ideal(Å)
1	L	151	GLY	C-O	7.91	1.36	1.23
3	A	145	TYR	CE1-CZ	-5.11	1.31	1.38

All (7) bond angle outliers are listed below:

Mol	Chain	Res	Type	Atoms	Z	Observed(^{o})	Ideal(^{o})
3	A	166	MET	CG-SD-CE	-6.50	89.81	100.20
2	H	56	GLY	N-CA-C	5.82	127.66	113.10
3	A	178	ASP	CB-CG-OD2	-5.63	113.23	118.30
2	H	35	ASP	CB-CG-OD1	5.36	123.12	118.30
3	A	156	ARG	NE-CZ-NH1	5.35	122.98	120.30
2	H	34	LEU	CB-CG-CD1	-5.30	101.99	111.00
3	A	156	ARG	NE-CZ-NH2	-5.19	117.71	120.30

CONFIDENTIAL VALIDATION REPORT

There are no chirality outliers.

All (1) planarity outliers are listed below:

Mol	Chain	Res	Type	Group
2	H	56	GLY	Peptide

5.2 Too-close contacts (i)

In the following table, the Non-H and H(model) columns list the number of non-hydrogen atoms and hydrogen atoms in the chain respectively. The H(added) column lists the number of hydrogen atoms added and optimized by MolProbity. The Clashes column lists the number of clashes within the asymmetric unit, whereas Symm-Clashes lists symmetry related clashes.

Mol	Chain	Non-H	H(model)	H(added)	Clashes	Symm-Clashes
1	L	1626	0	1555	10	0
2	H	1585	0	1532	15	0
3	A	848	0	788	5	0
4	H	5	0	0	0	0
5	A	18	0	0	0	0
5	H	20	0	0	0	0
5	L	25	0	0	0	0
All	All	4127	0	3875	30	0

The all-atom clashscore is defined as the number of clashes found per 1000 atoms (including hydrogen atoms). The all-atom clashscore for this structure is 4.

All (30) close contacts within the same asymmetric unit are listed below, sorted by their clash magnitude.

Atom-1	Atom-2	Interatomic distance (Å)	Clash overlap (Å)
1:L:1:GLN:N	1:L:1:GLN:OE1	1.72	1.21
2:H:33:ASN:HD21	2:H:50:ASN:HD22	1.31	0.78
2:H:158:SER:H	2:H:198:ASN:HD21	1.40	0.69
2:H:33:ASN:ND2	2:H:50:ASN:HD22	1.97	0.59
2:H:137:SER:N	2:H:187:SER:HG	2.00	0.59
2:H:179:LEU:C	2:H:179:LEU:HD12	2.26	0.57
1:L:1:GLN:N	1:L:1:GLN:CD	2.55	0.56
2:H:16:SER:O	2:H:86:LEU:HD12	2.06	0.55
3:A:178:ASP:O	3:A:182:ILE:HD12	2.07	0.54
2:H:17:SER:HA	2:H:86:LEU:CD1	2.38	0.54
2:H:30:THR:O	2:H:54:ASN:ND2	2.40	0.54
2:H:6:GLN:H	2:H:108:GLN:HE22	1.55	0.53
2:H:158:SER:H	2:H:198:ASN:ND2	2.05	0.53

Continued on next page...

CONFIDENTIAL VALIDATION REPORT

Continued from previous page...

Atom-1	Atom-2	Interatomic distance (Å)	Clash overlap (Å)
2:H:31:ASP:OD1	2:H:31:ASP:N	2.45	0.50
3:A:139:ILE:HG22	3:A:141:PHE:CE2	2.48	0.49
3:A:201:THR:O	3:A:205:MET:HG3	2.14	0.48
1:L:144:ASN:O	1:L:195:ALA:HA	2.14	0.48
1:L:119:PRO:HD3	1:L:131:VAL:HG22	1.95	0.48
3:A:164:ARG:HD3	3:A:168:GLU:OE1	2.15	0.47
2:H:17:SER:HA	2:H:86:LEU:HD12	1.98	0.45
1:L:166:ASP:O	1:L:170:SER:HA	2.16	0.44
1:L:61:PHE:CE2	1:L:74:ILE:HG12	2.53	0.43
2:H:156:TRP:CZ3	2:H:197:CYS:HB3	2.53	0.43
1:L:191:TYR:O	1:L:207:SER:HA	2.19	0.43
1:L:7:SER:HA	1:L:8:PRO:C	2.38	0.42
1:L:177:THR:HG22	1:L:179:THR:HG22	2.01	0.42
2:H:20:ILE:HG21	2:H:20:ILE:HD13	1.68	0.42
2:H:11:LEU:HB2	2:H:149:PRO:HG3	2.03	0.41
3:A:184:ILE:O	3:A:188:THR:HG23	2.21	0.40
1:L:35:TYR:N	1:L:35:TYR:CD1	2.90	0.40

There are no symmetry-related clashes.

5.3 Torsion angles (i)

5.3.1 Protein backbone (i)

In the following table, the Percentiles column shows the percent Ramachandran outliers of the chain as a percentile score with respect to all X-ray entries followed by that with respect to entries of similar resolution.

The Analysed column shows the number of residues for which the backbone conformation was analysed, and the total number of residues.

Mol	Chain	Analysed	Favoured	Allowed	Outliers	Percentiles	
1	L	210/212 (99%)	201 (96%)	9 (4%)	0	100	100
2	H	207/215 (96%)	193 (93%)	13 (6%)	1 (0%)	31	38
3	A	99/113 (88%)	99 (100%)	0	0	100	100
All	All	516/540 (96%)	493 (96%)	22 (4%)	1 (0%)	49	61

All (1) Ramachandran outliers are listed below:

CONFIDENTIAL VALIDATION REPORT

Mol	Chain	Res	Type
2	H	30	THR

5.3.2 Protein sidechains (i)

In the following table, the Percentiles column shows the percent sidechain outliers of the chain as a percentile score with respect to all X-ray entries followed by that with respect to entries of similar resolution.

The Analysed column shows the number of residues for which the sidechain conformation was analysed, and the total number of residues.

Mol	Chain	Analysed	Rotameric	Outliers	Percentiles	
1	L	185/185 (100%)	170 (92%)	15 (8%)	13	16
2	H	184/186 (99%)	165 (90%)	19 (10%)	8	9
3	A	95/102 (93%)	90 (95%)	5 (5%)	25	34
All	All	464/473 (98%)	425 (92%)	39 (8%)	12	14

All (39) residues with a non-rotameric sidechain are listed below:

Mol	Chain	Res	Type
1	L	1	GLN
1	L	27	SER
1	L	28	SER
1	L	39	SER
1	L	55	SER
1	L	125	THR
1	L	142	ASP
1	L	152	SER
1	L	154	ARG
1	L	164	ASP
1	L	167	SER
1	L	179	THR
1	L	181	THR
1	L	189	ASN
1	L	206	LYS
2	H	1	GLU
2	H	12	VAL
2	H	28	THR
2	H	31	ASP
2	H	34	LEU
2	H	44	THR
2	H	63	LYS

Continued on next page...

CONFIDENTIAL VALIDATION REPORT

Continued from previous page...

Mol	Chain	Res	Type
2	H	74	LYS
2	H	75[A]	SER
2	H	75[B]	SER
2	H	103	VAL
2	H	123	SER
2	H	161	LEU
2	H	171	VAL
2	H	179	LEU
2	H	197	CYS
2	H	198	ASN
2	H	206	THR
2	H	208	VAL
3	A	125	LEU
3	A	160	GLN
3	A	193	THR
3	A	203	VAL
3	A	208	ARG

Some sidechains can be flipped to improve hydrogen bonding and reduce clashes. All (8) such sidechains are listed below:

Mol	Chain	Res	Type
1	L	188	HIS
1	L	189	ASN
2	H	33	ASN
2	H	198	ASN
3	A	171	ASN
3	A	174	ASN
3	A	181	ASN
3	A	212	GLN

5.3.3 RNA (i)

There are no RNA molecules in this entry.

5.4 Non-standard residues in protein, DNA, RNA chains (i)

There are no non-standard protein/DNA/RNA residues in this entry.

CONFIDENTIAL VALIDATION REPORT

5.5 Carbohydrates (i)

There are no carbohydrates in this entry.

5.6 Ligand geometry (i)

1 ligand is modelled in this entry.

In the following table, the Counts columns list the number of bonds (or angles) for which Mogul statistics could be retrieved, the number of bonds (or angles) that are observed in the model and the number of bonds (or angles) that are defined in the Chemical Component Dictionary. The Link column lists molecule types, if any, to which the group is linked. The Z score for a bond length (or angle) is the number of standard deviations the observed value is removed from the expected value. A bond length (or angle) with $|Z| > 2$ is considered an outlier worth inspection. RMSZ is the root-mean-square of all Z scores of the bond lengths (or angles).

Mol	Type	Chain	Res	Link	Bond lengths			Bond angles		
					Counts	RMSZ	$\#\|Z\| > 2$	Counts	RMSZ	$\#\|Z\| > 2$
4	SO4	H	301	-	4,4,4	0.24	0	6,6,6	1.22	1 (16%)

There are no bond length outliers.

All (1) bond angle outliers are listed below:

Mol	Chain	Res	Type	Atoms	Z	Observed(^{o})	Ideal(^{o})
4	H	301	SO4	O4-S-O3	2.49	119.97	108.98

There are no chirality outliers.

There are no torsion outliers.

There are no ring outliers.

No monomer is involved in short contacts.

5.7 Other polymers (i)

There are no such residues in this entry.

5.8 Polymer linkage issues (i)

There are no chain breaks in this entry.

CONFIDENTIAL VALIDATION REPORT

6 Fit of model and data (i)

6.1 Protein, DNA and RNA chains (i)

In the following table, the column labelled '#RSRZ> 2' contains the number (and percentage) of RSRZ outliers, followed by percent RSRZ outliers for the chain as percentile scores relative to all X-ray entries and entries of similar resolution. The OWAB column contains the minimum, median, 95th percentile and maximum values of the occupancy-weighted average B-factor per residue. The column labelled 'Q< 0.9' lists the number of (and percentage) of residues with an average occupancy less than 0.9.

Mol	Chain	Analysed	<RSRZ>	#RSRZ>2			OWAB(Å^2)	Q<0.9
1	L	210/212 (99%)	-0.16	9 (4%)	35	42	18, 48, 95, 103	21 (10%)
2	H	208/215 (96%)	-0.06	4 (1%)	66	73	18, 48, 87, 111	16 (7%)
3	A	101/113 (89%)	-0.36	1 (0%)	82	86	23, 33, 73, 94	6 (5%)
All	All	519/540 (96%)	-0.16	14 (2%)	54	62	18, 43, 89, 111	43 (8%)

All (14) RSRZ outliers are listed below:

Mol	Chain	Res	Type	RSRZ
1	L	127	GLY	4.6
1	L	185	TYR	4.1
1	L	125	THR	3.3
2	H	26	ARG	3.2
2	H	158	SER	3.1
1	L	126	GLY	2.6
1	L	121	SER	2.5
3	A	193	THR	2.5
2	H	209	ASP	2.3
2	H	42	GLY	2.3
1	L	180	LEU	2.3
1	L	147	TRP	2.1
1	L	120	SER	2.1
1	L	149	ILE	2.0

6.2 Non-standard residues in protein, DNA, RNA chains (i)

There are no non-standard protein/DNA/RNA residues in this entry.

CONFIDENTIAL VALIDATION REPORT

6.3 Carbohydrates (i)

There are no carbohydrates in this entry.

6.4 Ligands (i)

In the following table, the Atoms column lists the number of modelled atoms in the group and the number defined in the chemical component dictionary. The B-factors column lists the minimum, median, 95th percentile and maximum values of B factors of atoms in the group. The column labelled 'Q< 0.9' lists the number of atoms with occupancy less than 0.9.

Mol	Type	Chain	Res	Atoms	RSCC	RSR	B-factors(Å^2)	Q<0.9
4	SO4	H	301	5/5	0.97	0.22	22,23,25,27	5

6.5 Other polymers (i)

There are no such residues in this entry.

CONFIDENTIAL VALIDATION REPORT
